# Blue Microbiology—Aquatic Microbial Resources for Sustainable Life on Earth

**DOI:** 10.3390/microorganisms11030808

**Published:** 2023-03-22

**Authors:** Chatragadda Ramesh, Laurent Dufossé

**Affiliations:** 1 Biological Oceanography Division, National Institute of Oceanography (CSIR-NIO), Dona Paula 403004, Goa, India; 2 Chemistry and Biotechnology of Natural Products, CHEMBIOPRO, Université de La Réunion, ESIROI Agroalimentaire, 15 Avenue René Cassin, CEDEX 9, F-97744 Saint-Denis, France

The exploration of the microbial world in research continues to be fascinating and unending in several aspects of taxonomy, genomics, evolution, and industrial applications [1]. Among numerous known microbes from different environments [1], aquatic microbes have attained significant demand for decades to address the benefits of aquatic microbes for societal issues, such as drugs to cure diseases and several other molecules as preservatives and additives [2,3,4]. In this context, to attract the current applications of aquatic microbes, the Special Issue “Blue Microbiology—Aquatic Microbial Resources for Sustainable Life on Earth” was proposed to cover the current trends in the exploration and utilization of new products from marine and fresh-water microbial biodiversity (archaea, bacteria, cyanobacteria, yeasts, and filamentous fungi) using known and new methods for the advancement of science and societal demands.

This Special Issue on “Blue Microbiology” aimed to cover the aspects of microbial ecology, molecular biology, and aquatic microbial bioprospecting. In this Special Issue, the latest techniques in marine microbial culture in the laboratory, screening of isolates for bioprospecting, and use of microreactors and miniaturized systems in bioprocessing development were reviewed in detail by Rodrigues and de Carvalho [5]. On the other hand, Chernogor et al. [6] published the work on the extraction, characterization, and identification of violet pigments violacein and deoxyviolacein isolated from *Janthinobacterium* sp. SLB01 strain. In this research, Chernogor et al. [6] isolated strain SLB01 from a diseased sponge *Lubomirskia baicalensis* collected from Lake Baikal, and reported the maximum violacein production and biofilm formation at 22 °C. They have inferred that violacein pigments can be useful in biotechnological applications. In terms of ecology, violacein production is suggested to impact the microbial communities assembled on sponges in Lake Baikal.

The importance of taxonomical identification of microbes up to the species level was emphasized by two research articles in this Special Issue. Lozano and collaborators reported the quality and reproducibility of tandem mass spectrometry proteotyping technique to identify microbes isolated from coastal water of the northwest Mediterranean Sea. Based on a single MS/MS analytical run, *Balneola vulgaris*, is identified for the first time and inferred that the workflow is effective in rapidly identifying microorganisms in addition to molecular characterization [7]. Another article published in this Special Issue by Ramesh et al. [8] detailed a whole genome-based workflow to identify undecylprodigiosin pigment-producing marine *Streptomyces* strain BSE6.1. Based on the distinctive genomic content variability, strain BSE6.1 was proposed as *Streptomyces prasanthi* sp. nov. On the other hand, Hurtado-McCormick et al. [9] provided a standardization toolbox based on the available 16S rRNA amplicon sequencing dataset to understand the blue carbon budget accumulation and return to the atmosphere. These articles suggest that identifying microorganisms up to species level using MS/MS technique and molecular characterization as reliable techniques. At the same time, 16S rRNA amplicon sequence data of microbes isolated from the specific environment can give a detailed understanding of carbon budget accumulation and return to the atmosphere.

The remarkable conclusions made in this Special Issue by the above authors suggest the importance of novel techniques for rapid taxonomical and biotechnological advancement. Under this Blue Microbiology Special Issue, particularly marine microbes with ecological and biotechnological applications, have been significantly highlighted due to their role in the ecosystem structure as well as the multifaceted benefits posed to society. There is no doubt that the exploration, exploitation, and utilization of aquatic microbes, particularly marine microbes, continue to get more demand in the current and future research for various biotechnological applications to address societal needs. In brief, this Special Issue of Blue Microbiology covered the taxonomical importance, biotechnological applications, and ecological roles of microbes for future endeavors.
